# Etiology of syncope in hospitalized patients

**Published:** 2015

**Authors:** Mehrdad Saravi, Alijan Ahmadi Ahangar, Mohammad Masood Hojati, Ebrahim Valinejad, Ahmad Senaat, Reza Sohrabnejad, Mohammad Reza Khosoosi Niaki

**Affiliations:** 1 Department of Cardiology, Babol University of Medical Sciences, Babol, Iran.; 2 Department of Neurology, Babol University of Medical Sciences, Babol, Iran.; 3 Babol Clinic Hospital, Babol, Iran.

**Keywords:** Etiology, Syncope, Head-up tilt test, Electrophysiologic study

## Abstract

**Background:**

Syncope is a common clinical problem which can be remarkably debilitating and associated with high health care costs. Syncope is a clinical syndrome with many potential causes. The aim of the study was to determine the etiologies of patients with syncope in the emergency department (ED) of a referral and general university hospital.

**Methods:**

One hundred sixty-five consecutive patients aged more than 18 years old with syncope were admitted to the emergency department of Ayatollah Rouhani Hospital. Initially organized, systematic approach included detailed medical history and structured questionnaires for history taking, physical examination, ECG and cardiac monitoring, cardiology and neurology were done. Advanced diagnostic tests were carried out if the etiology of syncope remained unexplained.

**Results:**

Out of the 165 patients who presented to the ED between February 2012 and February 2013, 124 had definition of syncope. The mean age of male patients was 59.5±19.8, 58. The etiology of syncope was diagnosed in 104 (83%) patients. Neurocardiogenic syncope was found in 36 (29.03%) patients, cardiac arrhythmias in 40 (32.25%) patients, and acute coronary syndrome in 8 (6.45%) patients. There are some infrequent etiologies like intracranial hemorrhage in 5 patients, aortic stenosis in 4 patients, hypertrophic cardiomyopathy and aortic dissection in 3 patients, Brugada and pulmonary embolism in 2 patients and carotid hypersensitivity in one patient.

**Conclusion:**

We found that cardiac arrhythmias and neurocardiogenic type are the frequent causes of syncope. In about one-sixth of the patients, no etiology was found. Approximately one-third of patients had traumatic syncope.

**S**yncope is a transient loss of consciousness (T-LOC) due to transient global cerebral hypoperfusion characterized by rapid onset, short duration, and spontaneous complete recovery (1), which is not compatible with other states of altered consciousness (seizure, coma, migraine, metabolic disorders including, hypoxic ventilation, hypoglycemia, epilepsy, transient ischemic attack, cataplexy, drop attack, somatization disorders) (2, 3).

 Syncope is a common clinical problem accounting for 3% of all emergency room visits and 1% to 6% of all hospital admissions (4-9). Syncope can be remarkably debilitating and associated with high health care costs; its true incidence is difficult to estimate due to variation in definition, differences in population prevalence and underreporting in the general population (10-12). Syncope, a clinical syndrome has many potential causes. The prognosis of a patient experiencing syncope varies from benign outcome to increased risk of mortality or sudden death, determined by the etiology of syncope and the presence of underlying diseases.

Owing to the fact that a definitive diagnosis often cannot be established immediately, hospital admission is frequently recommended as the "default" approach to ensure the patient's safety and an expedited evaluation (13, 14). They are frequently admitted to hospital to undergo various and expensive investigations many of which have a low diagnostic yield (15). Hospital care is costly while no studies have shown that clinical outcomes are improved by the in-patient practice approach (13). 

The physician should establish a confident causal diagnosis, assess prognostic implications, and provide appropriate advice to prevent recurrences (16). Thus, the etiological classification remains the basis for both risk stratification and subsequent clinical management. Syncope is a presenting symptom, and in itself is not a diagnosis. While most causes are benign and self-limiting not requiring extensive in-hospital evaluation, others are potentially severe (17, 18). An etiology, mechanism or predisposition to a specific etiology must be sought. This predisposition is not necessarily responsible for the spontaneous syncope in all cases. A causal relationship between a diagnostic abnormality and syncope in the specific patient is often only presumptive. Currently, most clinicians classify syncope on clinical grounds by attempting to ascertain its etiology. They then use this classification to guide further management (12, 19, 20, 21). 

The optimal evaluation of patients with syncope follows a risk-adapted diagnostic algorithm exclude life-threatening conditions and identify those with high risk for further deterioration, such as structural heart diseases requiring further diagnostic evaluation. Low risk patients can be discharged without further extensive diagnostic work-up (18, 22). We want to determine the etiology of syncope in this area because the underlying disorders and social backgrounds associated with syncope may differ from those encountered in others, especially western countries (23). 

## Methods

In total, 124 consecutive patients with syncope, aged more than 18 years old who referred to the emergency department of a university hospital, North of Iran (Babol University of Medical Sciences) between February 2012 and February 2013 were studied. Patients with loss of consciousness from reasons other than hypoperfusion of the brain (seizure, concussion and trauma, intoxication, hypoglycemia, psychogenic pseudosyncope, seizure, head injury) and patients who were unable to give either written or verbal informed consent were excluded from the study. Diagnostic evaluations of the patients were performed by resident physicians on duty in the emergency department. All patients underwent initial evaluation and clinical assessment, which included detailed history, physical examination, metabolic, and medication review. From 169 patients, 45 patients were excluded because of non- syncopal loss of consciousness after initial evaluation.

Diagnostic workflow was conducted (European Guidelines) with regard to inpatient services. Over the following 24 hours, consultant cardiologist and neurologist reviewed all data. The patients who were diagnosed after this stage were discharged or treated based on the syncope etiology. For the rest of patients depended on the presence or absence of organic heart disease, abnormal ECG and clinical features suggestive of arrhythmias (palpitation, family history of sudden cardiac death, and syncope during exercise), abnormal neurologic data; diagnostic tests were carried out like transthoracic echocardiography (TTE) heart monitoring in emergency room (12) and monitoring of 24-hour of rhythm (1, 120 tilt table test (1, 12) and electrophysiological study (EPS) (12), computerized tomography (CT) scan of brain, magnetic resonance imaging (MRI) of brain, electroencephalogram (EEG) (1, 24, 25). 

If the diagnosis was not determined after these evaluations and targeted tests, the results of diagnostic procedures (cardiology and neurology) were re-evaluated. Diagnosis of typical neurocardiogenic syncope was based on clinical history [syncope occurring during prolonged standing, instrumentation, stress, or fear with typical prodromal symptoms (diaphoresis, warmth, and nausea)] and the result of tilt-table test (12, 15). 

Statistical analysis was performed using SPSS Version 19.0 (SPSS Inc. Chicago, IL, USA). Data were presented as mean±standard deviation. Comparison of normally distributed continuous variables was performed using a paired student's t-test and χ^2^ test or the Fisher’s exact test were used for dichotomous variables. ANOVA test was used for multiple comparisons. 

## Results

Among the 165 patients, syncope was documented in 124 patients which 46.2% of them were men. The mean age of the patients was (55.5±17.2) years; 58 (46.7 %) patients were males (table 1). There was history of diabetes mellitus in 20 (19.2) patients; hypertension was reported in 35 (33.7) patients, coronary artery disease in 29 (27.8%) patients, and congestive heart failure in 13 (12.5%) patients. The syncopal episodes were associated with trauma in 35 (33.7%) patients, about 30% of patients were over 70 years old. 

**Table 1:** Baseline characteristics of 104 patients with syncope

**Variable **	**Mean±SD** **or number (%)**
Age (Year)	59.5±19.8
Male	58 (46.7)
Systemic hypertension	35 (33.7)
Diabetes Mellitus	20 (19.2)
Coronary artery disease	29 (27.8)
Congestive heart failure	13 (12.5)
Smoker	18 (14.5)
Trauma /injury during syncope	35 (33.7%)
Absence of prodromal symptoms	40 (32.2)

The most common warning symptoms were palpitation (37.5%), sweating (24%), and chest pain (18.3%). The most common position in our patients at the time of syncope was standing position (39.4%). Table 2 shows the various etiologies of syncope diagnosed during the hospital workup. 

**Table 2:** Etiology of syncope in 124 hospitalized patients

**Etiology **	**Number (%)**
Neurocardiogenic	36 (29.03)
Sick sinus syndrome	13 (10.48)
Advanced AV Block	15 (12.9)
Supraventricular tachyarrhythmia	5 (4.03)
Ventricular tachyarrhythmia	7 (5.64)
Acute coronary syndrome	8 (6.45)
Aortic stenosis	4 (3.22)
Hypertrophic cardiomyopathy	3 (2.41)
Carotid hypersensitivity	1 (0.80)
Brugada Syndrome	2 (1.61)
Aortic dissection	3 (2.41)
Pulmonary embolism	2 (1.61)
Intracranial hemorrhage	5 (4.03)
Unknown	20 (16.12)

Neurocardiogenic syncope was found in 36 (29.03%) patients, cardiac arrhythmias in 40 (32.25%) patients, and acute coronary syndrome in 8 (6.45%) patients. The etiology of syncope was not determined in 20 (16.12%) patients. In age distribution, the lower age related to neurocardiogenic was found in 90% of youth and sick sinus syndrome (SSS) and atrioventricular (AV) block and aortic stenosis were seen in more advanced age (78% over 65 years old). Three patients need aortic valve replacement with the diagnosis of severe aortic stenosis. Electrophysiological study (EPS) was done in 21 patients. 

Fifteen percent of patients were discharged after evaluation in the ED. ECG was abnormal in 59.8% of patients. Cranial computed tomography (129 patients) and MRI of brain and EEG were done for many patients, but these tests only rarely yielded abnormal findings 4.03%. 

## Discussion

We found that cardiac arrhythmias and neurocardiogenic type are frequent causes of syncope. We choose a classic approach in patients presenting with syncope according to standard guidelines, which was similar to other studies of etiology of syncope in hospital (1, 12, 26). One third of the patients had traumatic syncope. In about one-sixth of the patients, no etiology was found. While neurocardiogenic syncope was more frequent in youth, multiple causes are often present in the elderly. 

In the studies of Sule S et al. (15), Kossaify A et al. (27), Kenny RA (10), Khera AD et al. (22), Serrano LA et al. (26), Mitro P (12), Blanc J.J. (7), neurocardiogenic syncope was one of the major etiologies for syncope. Cardiac arrhythmias were found in 20% of patients in Sule et al.’s (15) series. Some research studies found that AV block and new ischemic changes are common and important in syncopal patients (3, 10, 12, 17, 21, 22, 28). Like our study, neurocardiogenic syncope is seen in younger age and cardiovascular diseases in older patients (7, 10, 12, 26). Traumatic syncope was reported in multiple series (7, 10, 12, 15, 25). Sule et al (15) reported unexplained syncope in one-fourth of the patients. The importance and need of a specialized evaluation unit such as emergency department-based syncope management unit is suggested by many researchers (1, 5, 10, 12, 14, 18, 23, 25, 29-32) For better evaluation of syncope, new techniques like implanted ECG loop recorder (ILR) and ambulatory blood pressure monitoring should be used (19). Further studies are required to identify unexplained syncope and which adult ED syncope patients require cardiac monitoring in the ED and the optimal duration of monitoring. The limitation of our observational type of research included the small sample size done in a single university hospital. 

**Conclusion**

Hence, a thoughtful organized, systematic approach including detailed medical history and structured questionnaires for history taking, careful initial examination and specialized syncope evaluation units are essential for the diagnosis and treatment of syncope. In this way, a substantial proportion of patients, the etiology will be evident after initial evaluation and no further investigation is required. Consequently, this holds a great promise in terms of reducing hospital admissions, reducing costs and improving outcomes for patients with syncope. 
